# What Is a Proving?

**Published:** 1888-06

**Authors:** P. P. Wells


### HOMŒOPATHIC PHYSICIAN.

A MONTHLY JOURNAL OF

HOMŒOPATHIC MATERIA MEDICA AND CLINICAL MEDICINE.

"If our school ever gives up the strict inductive method of Hahnemann, we are lost, and deserve only to be mentioned as a caricature in the history of medicine."—CONSTANTINE HERING.

Vol. VIII. JUNE, 1888. No. 6.

### WHAT IS A PROVING?

EDITORS HOMŒOPATHIC PHYSICIAN: I have just received a card, on which was printed the following: "Axiom—the effect of a poison is a proving." Also on another card, "Dear Doctor, please controvert or assent to the above. Yours, fraternally, * * *."

I neither "controverted" nor "assented" to the "axiom," but replied as follows: "A proving is the effect of a noxia on the human organism, and a truthful record in detail of the symptoms which declare the nature of such effect or effects; without the observation necessary for this detailed record any poisoning is *less* than a proving. And without such examination of this record as can decide the truth of this or that fact, if it be really the result of the noxia, there can be no such proving as makes an agent practically available for intelligent clinical duties under the homœopathic law."

Is it not a little remarkable, after all these years and what has been written, that there should be need of stating again what a *proving of a drug is?* It is still more so that one, who seems to have given much of time and attention to this work, should need to be told. But, sad as the truth is, it is only too apparent that most of those who have been engaged in this work of late years have entered upon it with no definite idea of the philosophical principles involved in the duty; or of the nature of the facts (symptoms) which are the legitimate objective of the proving.* Why is it that there is still so great ignorance of what constitutes a proving, and what a symptom, as appears in most of our modern provings? Why, indeed, but that these would-be provers don't know because they have not been taught! And why have they not been taught? Evidently because they have been so unfortunate as to fall under the tuition of those who did not themselves know. This ignorance on the part of the teacher is fully disclosed by the call from the desk on the pupils of a class to join the professor in re-proving of drugs, to refute or confirm the experience of his expert predecessors. This professor did not know enough to enable him to accept the testimony of those who did know perfectly. He would have that of neophytes before he would be satisfied. Alas! that those who pass among us as teachers and professors even, should be made of such stuff!

P. P. WELLS.

*In a late volume of the transactions of the American Institute appeared what purported to be "a proving" of an important drug, containing some hundreds of entries of what were probably supposed to be symptoms of that drug. Among them was this entry: "Got up in the morning and felt better!" *Ex uno omnes disce.* The other hundreds of entries were of little more value to the prescriber than this bit of twaddle. The ambitious young prover (for he is young), no doubt meant well, but he did not know.

